# Assessing Mental Distress in Victims of Work-Related Hand Injuries

**DOI:** 10.1192/j.eurpsy.2025.1875

**Published:** 2025-08-26

**Authors:** A. Haddar, I. Sellami, A. Hrairi, M. A. Ghrab, H. Zouari, A. Feki, M. Trigui, M. Hajjaji, M. L. Masmoudi, K. Jmal Hammami

**Affiliations:** 1 Occupational medicine, University Hospital Hedi Chaker; 2 LR/18/ES-28, University of Sfax; 3 Orthopedic, University Hospital Habib Bourguiba; 4 Rheumatology, University Hospital Hedi Chaker, Sfax, Tunisia

## Abstract

**Introduction:**

Work-related hand injuries can lead to significant physical impairments. However, their psychological impact is often underestimated. Mental distress, including anxiety, depression, and post-traumatic stress, may arise following such injuries, potentially hindering recovery and affecting both professional and personal life. Given the critical importance of hand function in daily tasks and occupational roles and the prevalence of hand injuries, addressing their psychological dimension is crucial.

**Objectives:**

This study aims to evaluate mental distress among individuals with work-related hand injuries (WRHI) and to identify the associated factors.

**Methods:**

We conducted a cross-sectional study, between January 2021 to December 2022, involving private sector workers, victims of WRHI. Data were collected using a pre-established questionnaire that covered sociodemographic and professional characteristics, details of the occupational accident, and medical information. Psychological distress was assessed using the Kessler Psychological Distress Scale (K6), and functional disability of the hand was evaluated using the Quick Disabilities of the Arm, Shoulder, and Hand (Quick DASH) score.

**Results:**

Our study included 136 workers, with a male-to-female ratio of 7.5. The average age was 41.2 ± 10.9 years, and the average body mass index (BMI) was 26.48 ± 4.4. Blue-collar workers made up 77.9% of the sample (n = 106). In 61.8% of cases, the injury involved the dominant hand. The median of time to return to work was 101.5 days (interquartile range (IQR) [67; 182.5]). Twenty-five participants (18.4%) reported discrimination at the workplace. The median of the Quick DASH score was 34.1 (IQR [13.6; 52.3]). The median K6 score was 8 (IQR [4; 15]), with 42 participants (30.9%) experiencing moderate psychological distress and 45 (33.1%) reporting severe psychological distress. The K6 score showed a significant correlation with the Quick DASH score (p < 0.001, r = 0.297), BMI (p = 0.031, r = 0.185), and time to return to work (p = 0.005, r = 0.241). Those with injuries to the dominant hand had higher K6 scores compared to those with non-dominant hand injuries (p = 0.043). Furthermore, the K6 score was associated with reports of workplace discrimination following the accident.

**Conclusions:**

Work-related hand injuries not only lead to physical impairments but also have a considerable psychological impact. These findings highlight the importance of comprehensive rehabilitation programs that address both the physical and psychological aspects of recovery to improve outcomes and support reintegration into the workforce.

**Disclosure of Interest:**

None Declared

